# Hygienic Treatment of Disease

**Published:** 1871-02

**Authors:** 


### HYGIENIC TREATMENT OF DISEASE.

Side by side with the use of medicine, and not second to it, is the so-called hygienic treatment of disease—the study and regulation of the vital forces. The influence that the physician exercises over the mind, and through the mind, over the body; the soothing or the stimulation of the nervous power; the calming of exaltation or the stirring up of apathy; the quieting of the over-busy brain or the spurring of the flagging will; the repose of over-used powers or the awaking of suspended vital functions; the subduing of the over-sensitive skin or the stimulating of it where wan, muddy, and lifeless; the limiting of supplies to the over-fed frame or the repair of the wasted body by the proper kinds of food and stimulants; the bringing into play, and so again into existence, muscle that had become wasted and paralyzed by disease; these are among the aims the physician seeks to accomplish, and these are among the means which he seeks to employ irrespectively, but by no means necessarily, without the use of medicine; these are among the agencies which you hold in your power in the treatment of disease, and that you, each of you, exercise daily in coping with the various forms of malady, of ailment, and of constitution.—*Lancet.*

